# Comparison of frailty in patients with nontuberculous mycobacterial lung disease and bronchiectasis: a prospective cohort study

**DOI:** 10.1186/s12890-022-02206-5

**Published:** 2022-11-03

**Authors:** Kohei Fujita, Yutaka Ito, Yuki Yamamoto, Osamu Kanai, Takuma Imakita, Issei Oi, Takanori Ito, Zentaro Saito, Tadashi Mio

**Affiliations:** 1 Division of Respiratory Medicine, Center for Respiratory Diseases, National Hospital Organization Kyoto Medical Center, 1-1, Fukakusa-Mukaihata, Fushimi, Kyoto, Japan; 2 Department of Respiratory Medicine, Allergy and Clinical Immunology, School of Medical Sciences, Nagoya City University, Nagoya, Japan; 3 HiLung Inc., Kyoto, Japan

**Keywords:** Nontuberculous mycobacteria, Mycobacterium avium complex, Collectin, Antimicrobial peptide, Surfactant protein-A, Surfactant protein-D, hCAP18/LL-37, Frail, Mental health

## Abstract

**Background:**

The incidence of nontuberculous mycobacterial lung disease (NTM-LD) peaks in middle- and old age groups, coinciding with senescence; thus, chronic infectious diseases can accelerate frailty and worsen mental health in the elderly. In this study, we aimed to compare the prevalence of physical and psychiatric frailty between patients with NTM-LD and bronchiectasis (BE).

**Methods:**

The Kihon Checklist Questionnaire (KCQ) was used to assess physical and psychiatric frailties and identify those at risk of requiring care among patients with newly diagnosed NTM-LD and BE. Additionally, the Hospital Anxiety and Depression Scale (HADS) scores and chronic inflammatory biomarkers of the alveolar region (surfactant protein [SP]-A, SP-D, and human cationic antibacterial protein [hCAP]/LL-37) were assessed and compared between NTM-LD and BE patients.

**Results:**

There were no significant differences in the background characteristics between the 33 NTM and 36 BE patients recruited. The KCQ revealed that the proportion of frail NTM patients at diagnosis was higher than that of frail BE patients (48.5% vs. 22.2%, *p* = 0.026). HADS scores were significantly higher in the NTM group than in the BE group (*p* < 0.01). Bronchoalveolar lavage fluid (BALF) hCAP/LL-37 and SP-D levels were significantly higher (*p *= 0.001), but serum hCAP/LL-37 levels were significantly lower in the NTM group than in the BE group (*p* = 0.023). However, there were no significant differences in the BALF and serum SP-D levels between the two groups.

**Conclusions:**

The number of frail NTM patients at diagnosis was significantly higher than that of frail BE patients. Biomarker analysis suggested that the former had more localized lung inflammation than the latter.

**Trial registration:**

This trial was prospectively registered in the Clinical Trials Registry (UMIN 000027652).

**Supplementary Information:**

The online version contains supplementary material available at 10.1186/s12890-022-02206-5.

## Background

Recent epidemiological studies have shown emerging concerns regarding nontuberculous mycobacterial (NTM) diseases worldwide [1–3]. NTM lung disease (NTM-LD) is difficult to treat, and treatment options are limited. In contrast to tuberculosis, NTM-LD follows a chronic course, and the timing of treatment induction remains controversial [4, 5].

The incidence of NTM-LD peaks in middle or older age groups, when changes due to senescence occur. With the emergence of an aging society, frailty in the elderly population has become a social issue. Several epidemiological studies have shown that the incidence of NTM disease is increasing, especially in older populations [3, 6]. The coexistence of frailty in patients diagnosed with chronic infectious diseases such as NTM is not well known. There are also no data comparing them to non-infected patients, such as those with bronchiectasis. Understanding the prevalence of frailty at the time of NTM diagnosis is important for clinicians managing such patients. There exists a close relationship between frailty and chronic inflammation caused by chronic infection [7], and it is important to understand the surrogate markers that reflect frailty.

Although several studies have evaluated the factors affecting susceptibility to NTM disease, the main causal factors for disease progression remain unknown [8–10]. With a focus on local inflammation, past studies have underscored the role of pulmonary surfactant protein (SP) A and D against the development of *Mycobacterium avium* complex (MAC) disease [11]. SP-A and SP-D belong to the collectin family and play several key roles in the innate immune system [11–13].

Antimicrobial peptides also play an important role in host-mycobacterial interactions [14, 15]. The human cationic antimicrobial protein and its C-terminal peptide LL-37 (hCAP18/LL-37) is the only known human cathelicidin, and has multiple functions and antimicrobial effects. Increasing hCAP/LL-37 enhances the host defense response against mycobacterial infections [16, 17]. Since the peak of NTM-LD incidence occurs in middle- and older age groups, which are often associated with frailty and senescence, we speculated that chronic inflammation caused by NTM-LD might affect disease severity and host frailty. To test this hypothesis, we conducted a prospective cohort study comparing frailty in patients with NTM-LD and bronchiectasis.

## Methods

### Patients

This prospective study was conducted at the National Hospital Organization Kyoto Medical Center (a 600-bed tertiary care hospital), Kyoto, Japan, between October 1, 2017, and May 31, 2020. This clinical trial was approved by the institutional review board (approval number:16–072) and was registered in the Clinical Trials Registry (UMIN 000027652: https://center6.umin.ac.jp/cgi-open-bin/ctr/ctr_view.cgi?recptno=R000027566 Date of first registration: 09/06/2017). All methods were performed in accordance with the relevant guidelines and regulations. Informed consent was obtained from all participants for their enrollment in this study. We prospectively recruited patients who had at least bronchiectasis on the CT images and were initially suspected of having NTM-LD, which was later confirmed by bronchoscopy. All participants then underwent bronchoalveolar lavage (BAL) examination. Depending on whether the BAL fluid culture met the NTM diagnostic criteria of the 2007 ATS/IDSA guidelines, [4] participants were assigned to one of two groups: those diagnosed with NTM-LD were assigned to the NTM group, and the others were assigned to the non-NTM bronchiectasis (BE) group.

### Chest CT findings

Chest CT findings were classified as nodular bronchiectatic (NB), fibrocavitary (FC), NB + FC, and unclassified patterns by the consensus of at least two chest physicians.

### Collectins and antimicrobial peptides

We evaluated serum and bronchoalveolar lavage fluid (BALF) collectins, hCAP/LL-37, and TNF-α levels using the following commercially available ELISA kits: Human Surfactant Protein-A ELISA (Bio Vendor, Brno, Czech Republic), human surfactant protein-D ELISA (LSBio Inc., Seattle, WA, USA), and human LL-37 ELISA kit (Hycult Biothec, Uden, The Netherlands). All procedures were performed according to the manufacturer’s instructions.

### Frailty assessment

We assessed participants’ physical and psychiatric frailties using the Kihon Checklist Questionnaire (KCQ) developed by the Japanese Ministry of Health, Labour and Welfare, to identify elderly individuals at risk of requiring care/support [18–20]. It has previously been validated for frailty screening in respiratory disease [21, 22]. We also assessed participants’ psychiatric health status using the Hospital Anxiety and Depression Scale (HADS) questionnaire [23].

The KCQ: a self-administered questionnaire consisting of 25 yes/no questions covering instrumental (three items) and social activities of daily living (four items), physical strength (five items), nutritional status (two items), oral function (three items), cognitive status (three items), and depression risk (five items). Frailty status was classified as robust [score: 0–3], pre-frail [score: 4–7], or frail [score: 8–25]. In this study, we defined frailty status as having pre-frail and frail scores.

HADS questionnaire: This questionnaire measures anxiety and depression in the general patient population. It comprises seven questions each for anxiety and depression, and takes two to five minutes to complete. Total scores of 8–10, 11–14 and 15–21 represent mild, moderate, and severe anxiety or depression, respectively.

The KCQ and HADS questionnaires are presented in Additional file 1.

### Statistical analyses

Statistical analyses were performed using SPSS version 26.0 (IBM Corp., Armonk, NY, USA). Continuous variables were compared between independent groups using the Mann–Whitney test (two groups) and Kruskal–Wallis test (multiple groups). Categorical variables were compared using the Fisher’s exact test. Paired data were analyzed using the paired Wilcoxon rank test. Results with a *P* value less than 0.05 were considered statistically significant.

## Results

### Clinical characteristics of patients with NTM-LD and bronchiectasis

**Fig. 1:**
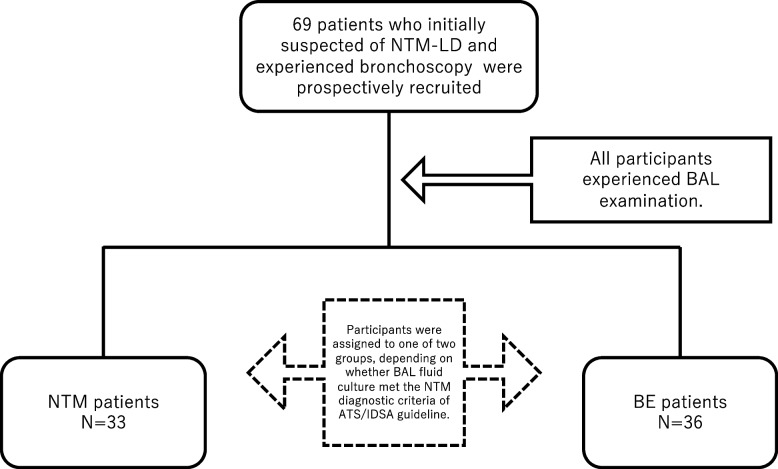
Flowchart of the study participants. All participants underwent BAL examination and were assigned to one of two groups, depending on whether BALF culture met the NTM diagnostic criteria. NTM-LD, nontuberculous mycobacterial lung disease; BE, bronchiectasis; BAL, bronchoalveolar lavage. ATS/IDSA = American Thoracic Society/Infectious Diseases Society of America

We prospectively recruited 69 patients who had undergone bronchoscopy. All patients had nodules and bronchiectasis on chest computed tomography (CT) and were initially suspected to have NTM-LD. Figure 1 shows the flowchart of study participant inclusion. After bronchoscopy, 33 patients met the diagnostic criteria for NTM; 36 patients did not meet the diagnostic criteria for NTM and were diagnosed with bronchiectasis.

Table 1 presents the patient characteristics. The mean age of patients with NTM and BE was 67.6 ± 10.8 and 68.6 ± 10.0 years, respectively. There were no significant differences in sex, BMI, smoking status, or underlying disease between patients with NTM and BE. Among the NTM patients, 15 and 9 patients were infected with *Mycobacterium avium* and *M. intracellulare*, respectively. Four patients were infected with *M. abscessus*. On radiological analysis, 24 patients with NTM showed an NB pattern. Six patients had a cavitary pattern (all patients had an NB + FC pattern). There were no significant differences in the thoracic abnormalities between patients with NTM and BE.

**Table 1:** Characteristics of patients with nontuberculous mycobacterial lung disease and bronchiectasis

Characteristics	Patients with NTM-LD	Patients with BE	*P* value
	*n* = 33	*n* = 36	
Age	67.6 ± 10.8	68.6 ± 10.0	0.7
Sex (female)	25 (75.8)	31 (86.1)	0.36
BMI	19.1 ± 2.4	20.3 ± 2.8	0.11
Smoking status	4 (12.1)	5 (13.9)	0.83
Underlying disease			
Asthma	0 (0.0)	3 (8.3)	0.24
COPD	1 (3.0)	2 (5.6)	0.99 <
History of tuberculosis	1 (3.0)	5 (13.9)	0.2
History of malignant disease	1 (3.0)	0 (0.0)	0.48
Diabetes mellitus	0 (0.0)	2 (5.6)	0.49
Autoimmune disease	1 (3.0)	5 (13.9)	0.2
Infected NTM strain			
*M. avium*	15 (45.5)	-	NE
*M. intracellulare*	9 (27.3)	-	NE
*M. avium* + *M. intracellulare*	3 (9.1)	-	NE
*M. abscessus*	4 (12.1)	-	NE
Others	2 (6.1)	-	NE
HRCT findings			
Nodular-bronchiectatic (NB)	24 (72.7)	-	NE
Combined NB + Fibro-cavitary	6 (18.2)	-	NE
Unclassified^a^	3 (9.1)	-	NE
Thoracic abnormality			
Scoliosis	10 (30.3)	8 (22.2)	0.58
Pectus excavatum	1 (3.0)	1 (2.8)	0.99 <

Data are presented as numbers (%) or means ± SD

*Abbreviations*: *NTM-LD* Nontuberculous mycobacterial lung disease, *BE* Bronchiectasis, *BMI* Body mass index, *COPD* Chronic obstructive pulmonary disease, *HRCT* High-resolution computed tomography, *NE* Not evaluated

^a^Unclassified: combined nodular-bronchiectatic + infiltrating shadow/ground glass opacity

### Prevalence of frailty in patients with NTM and BE

Table 2 shows the prevalence of frailty among study participants. Sixteen of the 33 (48.5%) patients with NTM had a frail status at diagnosis. In contrast, 8 of 36 (22.2%) BE patients had frailty at diagnosis. Thus, patients with NTM were more vulnerable to frailty than those with BE (*p* = 0.026).

**Table 2:** Prevalence of frailty and mental health instability in patients with NTM and BE

	Patients with NTM-LD	Patients with BE	*P* value
	*n* = 33	*n* = 36	
The Kihon Checklist Questionnaire			
Total score	4.2 ± 3.6	2.6 ± 2.2	0.075
Number of pre-frail status (score 4–7)	8 (24.2)	7 (19.4)	0.77
Number of frail status (score 8–25)	8 (24.2)	1 (2.8)	0.011
Number of frail patients (pre-frail + frail status)	16 (48.5)	8 (22.2)	0.026
HADS score			
HADS-A	5.4 ± 3.2	2.6 ± 2.0	< 0.01
Number of major-A (8 <)	6 (18.2)	1 (2.8)	0.049
HADS-D	6.8 ± 3.8	3.3 ± 3.2	< 0.01
Number of major-D (11 <)	4 (12.1)	1 (2.8)	0.19

Data are shown as number (%) or mean ± SD

*Abbreviations*: *NTM-LD* Nontuberculous mycobacterial lung disease, *BE* Bronchiectasis, *HADS* Hospital Anxiety and Depression Scale

### Mental health instability in patients with NTM and BE

Table 2 shows the participants’ mental health status. The anxiety and depression scores were significantly higher in patients with NTM than in those with BE. The number of patients with major anxiety and depression was also higher in the NTM group than in the BE group. Patients with NTM thus had greater mental health instability than patients with BE.

### Comparison of clinical characteristics in NTM patients with frail status and robust status

Table 3 shows the comparison of characteristics in NTM patients with frail status and robust status. NTM patients with frailty tend to have cavitary lesions more often, though this relationship was not statistically significant. All patients infected with *M. abscessus* had cavitary lesions on CT.

**Table 3:** Comparison of characteristics in NTM patients with frail status and robust status

	NTM patients with frail	NTM patients with robust	*P* value
	*n* = 16	*n* = 17	
Age	73.2 ± 8.1	62.4 ± 10.7	0.0089
Sex (female)	11 (68.8)	14 (82.4)	0.44
BMI	18.7 ± 2.6	19.4 ± 2.1	0.3
Smoking status	3 (18.8)	1 (5.9)	0.335
Underlying disease			
COPD	1 (6.3)	0 (0.0)	0.485
History of tuberculosis	0 (0.0)	1 (5.9)	0.99 <
History of malignant disease	0 (0.0)	1 (5.9)	0.99 <
Autoimmune disease	0 (0.0)	1 (5.9)	0.99 <
Infected NTM strain			
*M. avium*	5 (31.3)	10 (58.8)	-
*M. intracellulare*	5 (31.3)	4 (23.5)	-
*M. avium* + *M. intracellulare*	1 (6.3)	2 (11.8)	-
*M. abscessus*	4 (25.0)	0 (0.0)	-
Others	1 (6.3)	1 (5.9)	
HRCT findings			
Nodular-bronchiectatic (NB)	9 (56.3)	15 (88.2)	0.057
Combined NB + Fibro-cavitary	5 (31.3)	1 (5.9)	0.042
Unclassified^a^	2 (12.5)	1 (5.9)	0.601
Thoracic abnormality			
Scoliosis	6 (37.5)	4 (23.5)	0.47
Pectus excavatum	1 (6.3)	0 (0.0)	0.49

Data are shown as number (%) or mean ± SD

*Abbreviations*: *NTM* Nontuberculous mycobacteria, *BMI* Body mass index, *COPD* Chronic obstructive pulmonary disease, *HRCT* High-resolution computed tomography

^a^Unclassified: combined nodular-bronchiectatic + infiltrating shadow/ground glass opacity

### hCAP/LL-37 and pulmonary collectins levels in BALF and serum samples between patients with NTM and BE

**Fig. 2:**
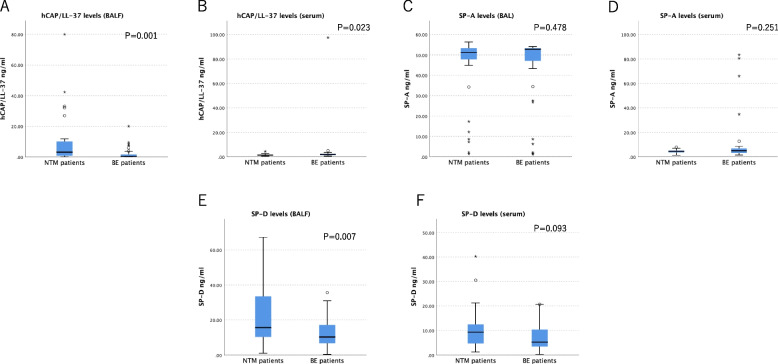
Profiles of BALF (**A**) and serum (**B**) concentration of hCAP/LL-37 between patients with NTM and BE patients and the profiles of BALF (**C**, **E**) and serum (**D**, **F**) concentration of SP-A and SP-D between the two groups. hCAP/LL-37, human cationic antimicrobial protein and its C-terminal peptide; BALF, bronchoalveolar lavage fluid; NTM, nontuberculous mycobacteria; BE, bronchiectasis; SP-A, surfactant protein A; SP-D, surfactant protein D. *n* = 33 (NTM patients) and 36 (BE patients)

Figure 2A-F shows the hCAP/LL-37 and pulmonary collectins (SP-A and SP-D) levels in BALF and serum samples from patients with NTM and BE. The concentration of hCAP/LL-37 in the BALF was significantly higher in the NTM group than in the BE group (*p* = 0.001). In contrast, the serum concentration of hCAP/LL-37 was significantly lower in patients with NTM than in patients with BE (*p* = 0.023).

SP-D concentration in BALF was significantly higher in the NTM group than in the BE group (*p* = 0.007). The serum SP-D concentration tended to be higher in NTM patients than in BE patients, but the difference was not significant. There were no significant differences in SP-A concentrations in the BALF and serum between the two groups.

### hCAP/LL-37 and pulmonary collectins levels in BALF and serum samples between frail and robust patients

**Fig. 3:**
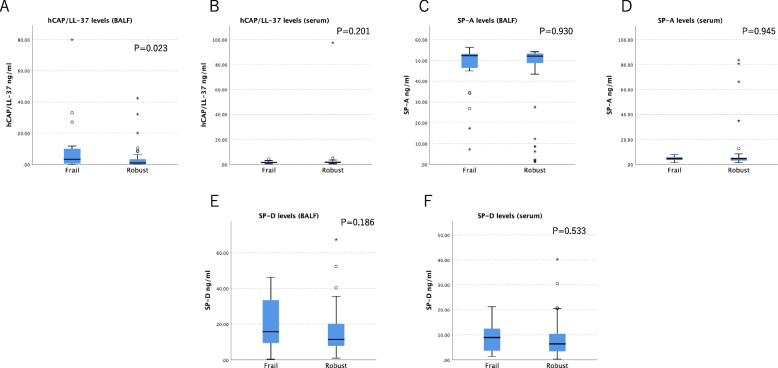
Profiles of BALF (**A**) and serum (**B**) concentration of hCAP/LL-37 between frail and robust patients and the profiles of BALF (**C**, **E**) and serum (**D**, **F**) concentration of SP-A and SP-D between frail and robust patients. hCAP/LL-37 = human cationic antimicrobial protein and its C-terminal 37 amino acid fragment; BALF, bronchoalveolar lavage fluid; SP-A, surfactant protein A; SP-D, surfactant protein D. *n* = 24 (frail patients) and 45 (robust patients)

Figure 3A-F shows the levels of hCAP/LL-37 and pulmonary collectins (SP-A and SP-D) in BALF and serum samples between frail and robust patients. The concentrations of hCAP/LL-37 and SP-D in BALF were significantly higher in frail patients than in robust patients (*p* = 0.01 and *p* = 0.007, respectively). In contrast, the serum concentration of hCAP/LL-37 was significantly lower in frail patients than that in robust patients (*p* = 0.023). There were no significant differences in the concentrations of SP-A and SP-D in the BALF and serum between frail and robust patients.

## Discussion

In this study, we found that patients with NTM were significantly more frail than patients with BE at the time of diagnosis. In addition, patients with NTM were found to have a more unstable mental status than those with BE. There are two reasons for selecting patients with bronchiectasis as controls in this study. First, it is to align the background factors with those of patients with NTM-LD. Patients with NTM-LD and patients with bronchiectasis have a similar background lung structure and are therefore assumed to have a similar susceptibility to the NTM disease. We consider it interesting and meaningful to compare patients with the same lung structure with and without NTM. Second, this study included patients who had undergone bronchoscopy. This inevitably means that patients with at least some lung structure disruption with bronchiectasis are included, because completely healthy subjects (who do not need to undergo bronchoscopy) are not included. We assessed frailty using a new measure called the KCQ. The KCQ is a simple frailty checklist approved by the Japanese Ministry of Health, Labour, and Welfare. It is widely used in Japan and has been used to screen for frailty in patients with respiratory diseases [19–22]. The advantages of this checklist are that it is simple to write, easy to interpret, and can be easily introduced in any medical institution.

Recently, two clinical studies have assessed health-related quality of life (HLQOL) in elderly patients with MAC-LD. In these two studies, two representative orthodox questionnaires, the St. George’s Respiratory Questionnaire (SGRQ) and SF-26, were used to assess HLQOL. Maekawa et al. showed that lung anomalies (consolidation/cavitary lesion/lung volume loss) were closely related to poor HLQOL [24]. In our study, NTM patients with frailty tended to have cavitary lesions more often than NTM patients who were robust. Asakura et al. revealed that elderly patients had reduced HLQOL, and that lung deterioration could reduce HLQOL. These previous studies suggest that patients with NTM-LD who are elderly or have cavitary lesions have a reduced HLQOL, which is an important risk factor for frailty [25]. Our study showed that patients with NTM-LD already had advanced frailty at the time of diagnosis. Although this study was based on patients with relatively mild NTM-LD diagnosed by bronchoscopy, the finding that even patients with relatively mild NTM-LD have an advanced frailty status at the time of diagnosis is noteworthy.

Chronic exposure to antigenic stress caused by clinical and subclinical infections may lead to chronic inflammation in the host, promoting immunosenescence and frailty [7, 26]. Chronic infections, such as Human Immunodeficiency Virus (HIV), are also known to promote host vulnerability [27]. Since NTM-LD is a long-term chronic infection lasting more than 10 years, it is not uncommon for it to be diagnosed more than 10 years since disease onset. We found that patients with NTM diseases were already more vulnerable to frailty than patients with BE at the time of diagnosis. Thus, when clinicians diagnose a patient with NTM-LD, they should be aware of the high probability that the patient is already frail.

Notably, all patients infected with *M. abscessus* were frail, as *M. abscessus* infection is more severe and refractory than MAC infection [28, 29]. Such differences in frailty between NTM strains should be noted. Unfortunately, it is not possible to describe subspecies of *M. abscessus* as subspecies classification is not possible at our institution.

Furthermore, we evaluated the serum and BALF levels of hCAP/LL-37 and collectins that are potential biomarkers of mycobacterial disease severity [16, 30]. We compared these biomarker concentrations in BALF and serum between NTM and BE patients, and between frail and robust patients. Interestingly, the concentration of hCAP/LL-37 in both NTM patients and frail patients was high in the local alveoli but low in the peripheral blood. This may reflect the difference between local and systemic responses to NTM infection. The results of our study suggest that local inflammation is more potent than its systemic counterpart and may accelerate frailty. In this study, the serum hCAP/LL-37 showed lower in the NTM-LD patients than in the bronchiectasis patients. The reversal results in these serum samples are the opposite of our initial expectations. The hypothesis is that hCAP/LL-37 acts defensively against bacterial infections, including NTM disease, and that the population that originally had a low systemic secretory response to hCAP/LL-37 is more susceptible to NTM disease as a result. Although not NTM disease, it has been reported, for example, that in leprae infections, serum hCAP/LL-37 is lower in the leprosy group than in healthy individuals, and even lower in the untreated group (more early infection) than in the treated group [31]. However, previous reports in patients with tuberculosis have shown a positive correlation between systemic hCAP/LL-37 secretion and disease severity, [32] so it is still susceptible to fluctuations depending on the progression of the disease, type of strains and the timing of blood sampling. The concentration of hCAP/LL-37 probably differs in the early, middle and long-term stages of infection, so the stage at which blood samples are taken is also an important factor. In any case, the results cannot be well explained by the present study alone. Further study will be needed.

Although hCAP/LL-37 has been shown to inhibit the development of mycobacteria, a recent study showed that hCAP/LL-37 is attenuated in its bactericidal activity by polar mycobacterial lipids of pathogenic mycobacteria, which causes resistance to hCAP/LL-37 [33, 34]. In patients with MAC-LD, the protective role of hCAP/LL-37 may be weaker compared to patients with tuberculosis and other mycobacterial infections.

The SP-D levels show a difference between the patients with NTM-LD and bronchiectasis, while SP-A levels do not. One putative answer to why SP-D differed from SP-A between patients with NTM and those with bronchiectasis may be due to the fact that SP-D and SP-A play distinct roles in NTM defense mechanism [11]. Previously, SP-A binds to *M. avium* via lipid, whereas SP-D binds to *M. avium* via lipoarabinomannan. Through this mechanism, SP-D is known to promote bacterial aggregation. The number of cells phagocytosed by macrophages is known to increase in the presence of SP-D, and SP-D may be significantly more secreted in the early stages of infection, such as the participants in the current study, to promote bacterial aggregation.

No relationship was noted between pulmonary collectins (SP-A, SP-D) and disease severity, but there was a negative relationship between frailty status at diagnosis and disease severity. This is evidence that frailty is more reflective of a patient's condition than serum biomarkers, suggesting that frailty assessment is important in the management of patient care. Patients with cavitary lesions on CT were more likely to be frail. Although the small number of patients in this study did not reach statistical significance, lung destruction on CT seems to be an important indicator of frailty.

This study had several limitations. First, we relied exclusively on questionnaires to assess frailty. Therefore, there may have been a discrepancy between actual physical vulnerability and the assessment of vulnerability because it is not based on specific physical findings such as bone mineral density or grip strength loss. On the other hand, the advantage of this study is that it demonstrates that vulnerability can be screened by simple, non-invasive questionnaires. Second, this was a cross-sectional analysis that only examined frailty status at diagnosis. It did not examine how frailty changes with or without therapeutic interventions or the changes over time. Finally, the biomarkers measured in this study are only a small fraction of the indicators reflecting systemic inflammation; many other biomarkers exist. Not all causes of inflammation can be explained by the biomarkers measured in this study.

## Conclusions

In conclusion, we evaluated the prevalence of frailty in patients with NTM-LD and bronchiectasis. Patients with NTM-LD suffer from a more frail state compared to patients with BE, and biomarker analysis of serum and BALF suggested that the former had more localized lung inflammation than the latter.

## Supplementary Information

**Additional file 1. **TheKihon Checklist Questionnaire.

## Data Availability

The data used/analyzed this study can be provided on reasonable request, after ethical committee approval. Please contact the corresponding author if necessary.

## Abbreviations

BAL: Bronchoalveolar lavage
BALF: Bronchoalveolar lavage fluid
BE: Bronchiectasis
CT: Computed tomography
ELISA: Enzyme-linked immunosorbent assay
FC: Fibrocavitary (lesion)
HADS: Hospital Anxiety and Depression Scale
HLQOL: Health-related quality of life
KCQ: Kihon Checklist Questionnaire
MAC: *Mycobacterium avium* Complex
NB: Nodular bronchiectatic (lesion)
NTM: Non-tuberculous mycobacteria
NTM-LD: NTM lung disease
SP: Surfactant protein
