# Supplementary material for: Comparison of frailty in patients with nontuberculous mycobacterial lung disease and bronchiectasis: a prospective cohort study
Source: BMC Pulm Med. 2022 Nov 3;22:395. doi: 10.1186/s12890-022-02206-5 (PMC9632157; doi:10.1186/s12890-022-02206-5)
Supplement: Supplementary file 1 — Additional file 1. TheKihon Checklist Questionnaire. [file 12890_2022_2206_MOESM1_ESM.docx]

The Kihon Checklist Questionnaire

| **No** | **Questions** | **Answer** | |
| --- | --- | --- | --- |
| 1 | Do you go out by bus or train by yourself? | Yes | No |
| 2 | Do you go shopping to buy daily necessities by yourself? | Yes | No |
| 3 | Do you manage your own deposits and savings at the bank? | Yes | No |
| 4 | Do you sometimes visit your friends? | Yes | No |
| 5 | Do your family or friends turn to you for advice? | Yes | No |
| 6 | Do you normally climb stairs without using handrail or wall for support? | Yes | No |
| 7 | Do you normally stand up from a chair without any aids? | Yes | No |
| 8 | Do you normally walk continuously for 15 minutes? | Yes | No |
| 9 | Have you experienced a fall in the past year? | Yes | No |
| 10 | Do you have a fear of falling while walking? | Yes | No |
| 11 | Have you lost 2kg or more in the past 6 months? | Yes | No |
| 12 | Height: cm, Weight: kg, BMI: kg/m2 If BMI is less than 18.5, this item is Yes | Yes | No |
| 13 | Do you have any difficulties eating tough foods compared to 6 months ago? | Yes | No |
| 14 | Have you choked on your tea or soup recently? | Yes | No |
| 15 | Do you often experience having a dry mouth? | Yes | No |
| 16 | Do you go out at least once a week? | Yes | No |
| 17 | Do you go out less frequently compared to last year? | Yes | No |
| 18 | Do your family or your friends point out your memory loss?  e.g."You ask the same question over and over again." | Yes | No |
| 19 | Do you make a call by looking up phone numbers? | Yes | No |
| 20 | Do you find yourself not knowing today’s date? | Yes | No |
| 21 | In the last 2 weeks have you felt a lack of fulfillment in your daily life? | Yes | No |
| 22 | In the last 2 weeks have you felt a lack of joy when doing the things you used to enjoy? | Yes | No |
| 23 | In the last 2 weeks have you felt difficulty in doing what you could do easily before? | Yes | No |
| 24 | In the last 2 weeks have you felt helpless? | Yes | No |
| 25 | In the last 2 weeks have you felt tired without a reason? | Yes | No |

Hospital Anxiety and Depression Scale (HADS) questionnaire

| D | A |  | D | A |  |
| --- | --- | --- | --- | --- | --- |
|  |  | **I feel tense or 'wound up':** |  |  | **I feel as if I am slowed down:** |
|  | 3 | Most of the time | 3 |  | Nearly all the time |
|  | 2 | A lot of the time | 2 |  | Very often |
|  | 2 | From time to time, occasionally | 1 |  | Sometimes |
|  | 0 | Not at all | 0 |  | Not at all |
|  |  |  |  |  |  |
|  |  | **I still enjoy the things I used to enjoy:** |  |  | **I get a sort of frightened feeling like 'butterflies' in the stomach:** |
| 0 |  | Definitely as much |  | 0 | Not at all |
| 1 |  | Not quite so much |  | 1 | Occasionally |
| 2 |  | Only a little |  | 2 | Quite Often |
| 3 |  | Hardly at all |  | 3 | Very Often |
|  |  |  |  |  |  |
|  |  | **I get a sort of frightened feeling as if something awful is about to happen:** |  |  | **I have lost interest in my appearance:** |
|  | 3 | Very definitely and quite badly | 3 |  | Definitely |
|  | 2 | Yes, but not too badly | 2 |  | I don't take as much care as I should |
|  | 1 | A little, but it doesn't worry me | 1 |  | I may not take quite as much care |
|  | 0 | Not at all | 0 |  | I take just as much care as ever |
|  |  |  |  |  |  |
|  |  | **I can laugh and see the funny side of things:** |  |  | **I feel restless as I have to be on the move:** |
| 0 |  | As much as I always could |  | 3 | Very much indeed |
| 1 |  | Not quite so much now |  | 2 | Quite a lot |
| 2 |  | Definitely not so much now |  | 1 | Not very much |
| 3 |  | Not at all |  | 0 | Not at all |
|  |  |  |  |  |  |
|  |  | **Worrying thoughts go through my mind:** |  |  | **I look forward with enjoyment to things:** |
|  | 3 | A great deal of the time | 0 |  | As much as I ever did |
|  | 2 | A lot of the time | 1 |  | Rather less than I used to |
|  | 1 | From time to time, but not too often | 2 |  | Definitely less than I used to |
|  | 0 | Only occasionally | 3 |  | Hardly at all |
|  |  |  |  |  |  |
|  |  | **I feel cheerful:** |  |  | **I get sudden feelings of panic:** |
| 3 |  | Not at all |  | 3 | Very often indeed |
| 2 |  | Not often |  | 2 | Quite often |
| 1 |  | Sometimes |  | 1 | Not very often |
| 0 |  | Most of the time |  | 0 | Not at all |
|  |  |  |  |  |  |
|  |  | **I can sit at ease and feel relaxed:** |  |  | **I can enjoy a good book or radio or TV program:** |
|  | 0 | Definitely | 0 |  | Often |
|  | 1 | Usually | 1 |  | Sometimes |
|  | 2 | Not Often | 2 |  | Not often |
|  | 3 | Not at all | 3 |  | Very seldom |

Please check you have answered all the questions

Scoring:

Total score: Depression (D) Anxiety (A)

0-7 = normal

8-10 = Borderline abnormal

11-21 = Abnormal
